# Extracellular matrix, regional heterogeneity of the aorta, and aortic aneurysm

**DOI:** 10.1038/s12276-019-0286-3

**Published:** 2019-12-19

**Authors:** Sayantan Jana, Mei Hu, Mengcheng Shen, Zamaneh Kassiri

**Affiliations:** 1 Department of Physiology, Cardiovascular Research Center, University of Alberta, Edmonton, AB Canada; 2 Stanford Cardiovascular Institute, Stanford University School of Medicine, Stanford, CA USA

**Keywords:** Experimental models of disease, Cardiovascular biology

## Abstract

Aortic aneurysm is an asymptomatic disease with dire outcomes if undiagnosed. Aortic aneurysm rupture is a significant cause of death worldwide. To date, surgical repair or endovascular repair (EVAR) is the only effective treatment for aortic aneurysm, as no pharmacological treatment has been found effective. Aortic aneurysm, a focal dilation of the aorta, can be formed in the thoracic (TAA) or the abdominal (AAA) region; however, our understanding as to what determines the site of aneurysm formation remains quite limited. The extracellular matrix (ECM) is the noncellular component of the aortic wall, that in addition to providing structural support, regulates bioavailability of an array of growth factors and cytokines, thereby influencing cell function and behavior that ultimately determine physiological or pathological remodeling of the aortic wall. Here, we provide an overview of the ECM proteins that have been reported to be involved in aortic aneurysm formation in humans or animal models, and the experimental models for TAA and AAA and the link to ECM manipulations. We also provide a comparative analysis, where data available, between TAA and AAA, and how aberrant ECM proteolysis versus disrupted synthesis may determine the site of aneurysm formation.

## Aortic aneurysm: Different locations, different causes

A review of aneurysm formation, swelling in blood vessel, in the aorta, examines distinctions between two forms of the condition and the role of proteins in the extracellular matrix which surrounds cells of the arterial wall. Rupture of aneurysms in the aorta, the body’s main artery, is a major cause of death. Researchers led by Zamaneh Kassiri at the University of Alberta, Edmonton, Canada, emphasize that aneurysms in the thoracic and abdominal regions of the aorta are distinct conditions with crucial differences in their causes. Disrupted production and assembly of the extracellular matrix and its proteins may underlie thoracic aneurysm formation. Factors triggering the degradation of extracellular matrix proteins may be more significant in abdominal aneurysms. Understanding the differing molecular mechanisms involved could help address the current lack of effective drug treatments for these dangerous conditions.

## Introduction

Aortic aneurysm is morphologically defined as a focal dilation and structural degradation of the aorta, which can occur at different sites along the aortic tree. Pathologically, formation and progression of an aortic aneurysm is driven by dysregulated cellular and acellular events. While thorough reviews on the cellular mechanisms and current medical management of thoracic aortic aneurysm (TAA)^1,2^ and abdominal aortic aneurysm (AAA)^2–4^ have been published elsewhere, this review focuses on the role of the aortic extracellular matrix (ECM) and the proteases and their inhibitors that maintain the integrity and homeostasis of the ECM in the pathogenesis of TAA or AAA in humans and animal models.

## Aortic wall structure and the ECM

**Fig. 1:**
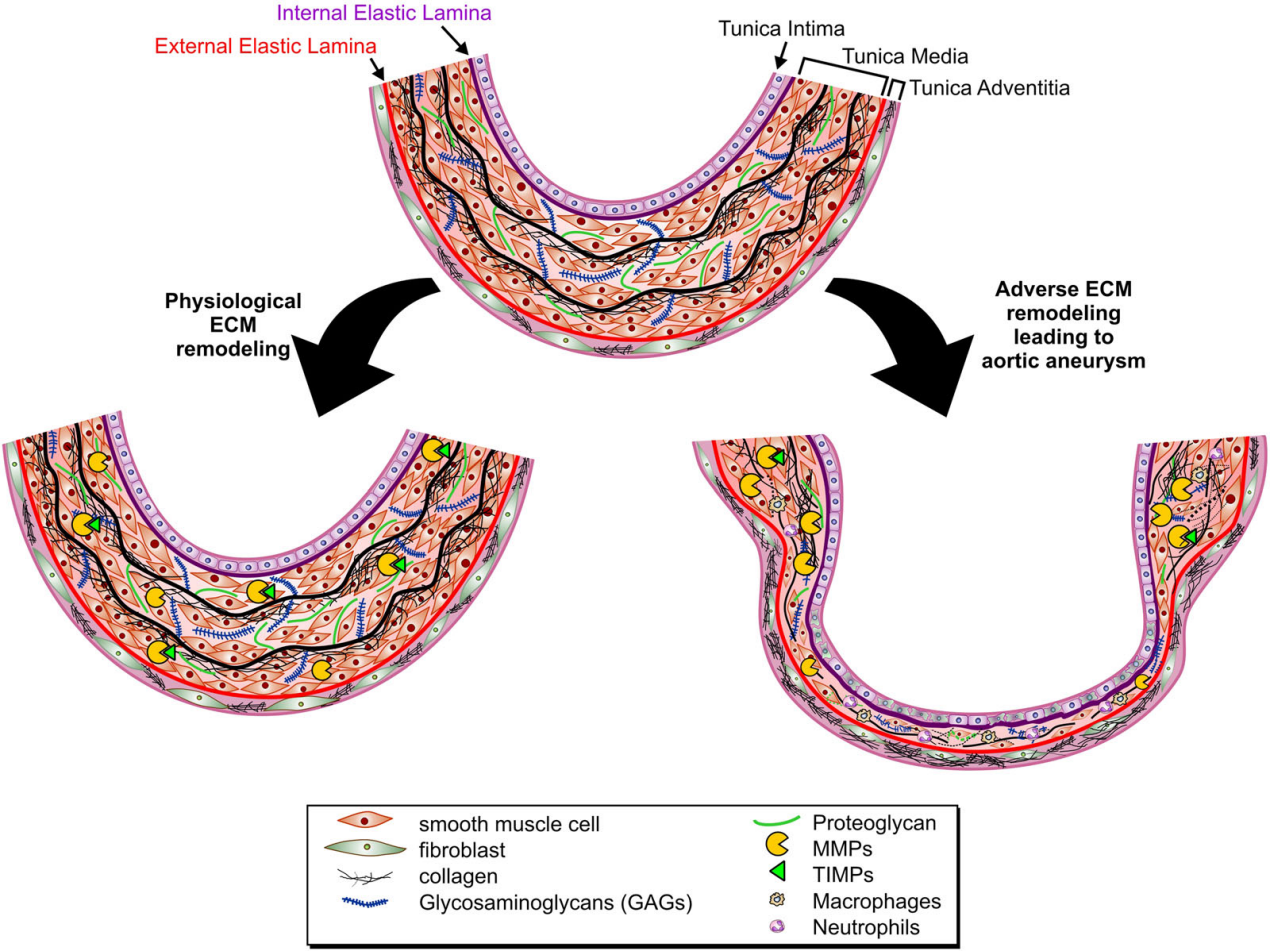
Cross section of the aortic wall and its remodeling during physiological versus pathological remodeling. The tunica media is separated from the tunica intima by the internal elastic lamina, and from the tunica adventitia by the external elastic lamina. Intermittent layers of elastic fiber and smooth muscle cells make up the tunica media, while this region is also rich in proteoglycans and glycoproteins. During physiological remodeling, degradation of ECM proteins by MMPs (and other proteases) is balanced by newly synthesized ECM proteins, while tissue inhibitors of proteases (TIMPs) keep the proteolytic activity of MMPs under check. Excess ECM degradation, or impaired ECM renewal synthesis, along with smooth muscle cell death, lead to adverse ECM remodeling as observed in aortic aneurysm

The aorta is the largest conduit artery in the body. Owing to its extraordinary ability to expand and recoil, the aorta also serves as a reservoir that transforms the highly pressured and pulsatile heart output into a flow of moderate fluctuations. The composition and three-dimensional organization of its ECM is critical for optimal physiological functions of the aorta. The aortic wall comprises an intricate arrangement of endothelial cells (ECs), smooth muscle cells (SMCs), fibroblasts (FBs), and ECM proteins in three layers: tunica intima, tunica media, and tunica adventitia (Fig. 1). The tunica intima consists of a single layer of ECs that lines the lumen of the blood vessel, and is anchored to the underlying basement membrane, a highly specialized ECM network consisting primarily of laminin, collagen type IV, fibronectin, perlecan, and heparan sulfate proteoglycans^5^. This basement membrane also plays a pivotal role in signaling events that regulate EC migration, invasion, proliferation, and survival^6^. The basement membrane together with the internal elastic lamina (IEL) serves as an interface between the tunica intima and tunica media. IEL is a thin sheet of elastic fiber with a variable number of fenestrations that facilitate diffusion of molecules through the tunica intima to the tunica media^7^. The tunica media consists of concentric elastic lamellae with circumferentially oriented SMCs. This layer is rich in ECM proteins, such as proteoglycans (PGs), glycoproteins, glycosaminoglycans (GAGs), as well as collagen. The intermittent layers of elastic fibers and SMCs in this layer provide the compliance and recoil properties of the aortic wall^8^. The medial layer of the aortic wall is the most variable of the tunics among different arteries. The differences in ECM composition in this layer account for the main variations in the function and properties of the arterial tree. The external elastic lamina (EEL) forms a boundary between the tunica media and tunica adventitia, the outermost layer of the aortic wall that is rich in collagen, FBs, and elastic fiber, and provides the tensile strength of the aortic wall. This collagen-rich tunica also harbors innervations, lymphatics, and the vasa vasorum, which are responsible for the high susceptibility of the adventitial layer to vascular inflammation.

The ECM is a key constituent of the vascular wall. SMCs and the FBs in the aortic wall synthesize and organize a complex ECM structure that defines the passive mechanical behavior of the large, elastic arteries such as the aorta. Arterial ECM is primarily composed of elastin, collagen, proteoglycans (PGs), and glycoproteins. Elastic fibers and fibrillar collagen, comprising ~50% of the dry weight of larger arteries^9^, are the predominant ECM components in the aortic wall and almost entirely define the mechanical properties of the aorta. Elastic fibers provide the expandability and recoil properties, while fibrillar collagens (predominantly collagen type I and type III) are responsible for the tensile strength of the aortic wall to withstand the high pressure of blood pumped by the heart^10^. In addition to their mechanical properties of the aortic wall, elastic fibers and fibrillar collagens can directly interact with various integrins and other proteins to modulate the adhesion, proliferation, and migration of SMCs^11–14^. GAGs consist of linear chains of repeating disaccharides and are highly negatively charged, which allow them to sequester water. Hydrated GAGs occupy a large volume of the extracellular space and cushion-compressive forces on cells. Among the four primary classes of GAGs, hyaluronan, chondroitin sulfate, heparan sulfate, and keratan sulfate, the first three are present in the aortic wall^15,16^. PGs are proteins with covalently attached GAG chains that provide mechanical support and mediate signaling responses for cells in the aortic wall. Versican, which is rich in chondroitin sulfate, is among the principal proteoglycans present in the aorta^17^. Among small leucine-rich proteoglycans (SLRP), biglycan and decorin are reported to promote collagen fibrinogenesis in the aorta^18,19^. Thrombospondins, tenascins, and fibronectin are also common glycoproteins present in the aorta^20,21^.

## Regional heterogeneity of the aortic wall

Morphology, structural composition, and mechanical properties of the aortic wall vary in the ascending and along the descending regions^22^ to ensure optimal mechanical operation^23^. The tunica intima accounts for <1% of the total wall thickness and remains uniform along the aorta, whereas the thickness of tunica adventitia is negligible in the thoracic aorta but increases considerably along the abdominal aorta^24^. The medial layer exhibits the greatest regional variations and diversitise along the aorta. The thickness of tunica media and the aortic lumen size steadily decrease from the ascending to the descending aorta, such that the wall thickness-to-lumen diameter ratio remains constant throughout the aorta. In the thoracic aorta, elastin occupies a higher proportion of the tunica media compared with that in the abdominal aorta. The amount of elastin decreases with distance from the heart, as less pulse dampening is required in the distal aorta. Meanwhile, the amount of collagen, which provides strength and limits stretch at high pressure, remains approximately constant with distance from the heart^25^. Therefore, the elastin-to-collagen ratio is lower in the abdominal compared with the thoracic aorta. In addition, there is a linear relationship between the number of lamellar units and the tensional forces within the aortic wall, and the number of lamellar units decreases from the thoracic to the abdominal aorta^10^.

## Physiological remodeling of the ECM

The ECM composition and integrity are key determinants of the physical characteristics of the aortic wall. Vascular ECM undergoes continuous physiological remodeling, whereby the existing ECM proteins are proteolytically degraded to be replaced by newly synthesized proteins. Several protease families are involved in this process. Matrix metalloproteinases (MMPs) are the most explored ECM-degrading proteinases^26^, while other metalloproteinases such as ADAMs (a disintegrin and metalloproteinases), ADAM-TSs (ADAMs with thrombospondin motifs), or serine/cysteine proteases, including cathepsins and granzymes, can also contribute to this process.

MMPs are a family of zinc-containing calcium-dependent endopeptidases that are best known for their ECM-degrading function, although they also contribute to a number of other cellular events^26^. To date, 23 human MMPs have been identified, which are classified into six groups based on their substrate specificities: collagenase, gelatinase, stromelysin, matrilysin, membrane-type MMPs, and others. MMPs are mostly secreted as inactive zymogens and are activated by proteolytic removal of their N-terminal pro-domain. The proteolytic activity of MMPs is tightly regulated in vivo by their endogenous inhibitors, tissue inhibitor of metalloproteinases (TIMPs)^27,28^. Other MMP inhibitors, such as α2-macroglobulin, inhibit MMPs in the plasma and liquid phase by irreversibly removing them through endocytosis^29^. The four members of the TIMP family (TIMP-1, −2, −3, and −4) can collectively inhibit all MMPs and a number of ADAMs and ADAM-TSs^30^ through a covalent bond between their N terminus and the catalytic domain of the proteinase in a 1:1 stoichiometric ratio. In addition to inhibiting metalloproteinases, TIMPs play a number of MMP-independent functions^31–34^. Sustained balance in proteolytic turnover of the ECM proteins is critical in maintaining the structural and functional integrity of the aortic wall. Cathepsins, a family of serine, cysteine, and aspartyl proteases are lysosomal proteases that also possess extracellular functions and can cleave multiple ECM components, including fibronectin, laminin, and elastin, and a number of non-ECM components^35^. Granzymes are serine proteases that contribute to aortic remodeling and have been the subject of a recent review^36^.

## Aortic aneurysm

Aortic aneurysm is characterized by a localized irreversible dilatation of the aortic lumen by ≥50% of its original diameter. The dilatation is induced by aberrant and adverse remodeling of the aortic wall, including endothelial damage in the intima, SMC loss and ECM degradation in the media, and ECM degradation in the adventitia. Aortic aneurysm affects all three layers of the blood vessel, while any dilation of the aorta that is limited to the outer wall is considered a false aneurysm or pseudo aneurysm^37^. Aortic aneurysm is asymptomatic, but can progress, gradually leading to aortic dissection or rupture, and as such is associated with critical morbidity and mortality in the aging population and accounts for 1–2% of total death in developed countries^38^. Although TAA and AAA display similar physical appearances, they each demonstrate distinct etiologies, pathological features, epidemiology, and risk factors.

## Abdominal aortic aneurysm (AAA)

AAA represents dilation of the aorta in any portion of the infra-diaphragmatic aorta, including the most common site of AAA formation, the infrarenal aorta. Depending on the degree of dilatation, AAA severity is classified as small (<55 mm) or large (≥55 mm). In the latter case, the patient will be considered eligible for surgical repair^39^. AAA is the 12–15^th^ leading cause of death in the aged population, mostly males, over the age of 65 in many developed countries^4^. Almost 1–2% of all 65-year-old males have AAA, whereas 0.5% of women at 70 years of age are affected with AAA^40,41^. The overall mortality due to AAA rupture has been reported to be 65–85%^38^. However, AAA-related mortalities in both male and female populations are declining in developed countries^42^, with the United States and the United Kingdom showing the fastest decline in AAA mortalities in male (6.7–6.2% per year) and female (3.9–4% per year) populations. This was found to closely correlate with the decline in smoking prevalence in these countries. On the other hand, in many European countries, including Romania and Hungary, AAA mortalities have increased by 1.7–2.7% for males and 1–3.5% for females annually^42^.

AAA is a complex multifactorial disease that is affected by both environmental and genetic factors. AAA incidences depend on age and sex, with a higher prevalence in males over the age of 55 compared with females of the same age range (6:1)^43^. However, AAA appears more detrimental in females, as they usually experience a higher risk of a small aneurysm rupture and less eligibility for standard surgical repair, because of the more complicated aneurysm morphology^44,45^. Other risk factors include smoking, a family history of aneurysm^46^, the presence of other diseases such as coronary heart disease, hyperlipidemia and atherosclerosis, hypertension, as well as acute or chronic infection^4,38^. Tobacco smokers have 3–4-fold greater risk of developing AAA than nonsmokers^47,48^. The link between hypertension and AAA is paradoxical, since hypertension has been shown to increase the risk of AAA by 30–40%; however, antihypertensive medication further increased the risk by 70–80%^47^. More recently, a randomized placebo-controlled trial reported that antihypertensive medications did not impact AAA growth, despite effective lowering of BP^49^. Interestingly, patients with diabetes mellitus have shown decreased prevalence or progression of AAA^50^, which could be linked to the effects of antidiabetic medications. Hypercholesterolemia, increased body mass index, lack of exercise, and cardiovascular diseases were found to be associative factors for higher incidences of AAAs in Swedish male population^51^. Although atherosclerosis shows a close association with AAA, multiple studies have demonstrated the lack of a causal role for atherosclerosis in the initiation of AAA^52–54^.

## Thoracic aortic aneurysm (TAA)

TAA represents dilation of one or more aortic segments above the diaphragm, including the aortic root, ascending aorta, aortic arch, and descending aorta. Approximately 60% of TAA occurs in the aortic root or the ascending aorta, 10% in the aortic arch, 40% in the descending aorta, and 10% in the thoraco-abdominal aorta^55^. About 13% of patients having aortic aneurysm develop multiple aneurysms, and ~20% of them can have a large TAA along with an AAA^56^. The prevalence of TAA is much less than that of AAA^57,58^, but the prognosis of TAA is more dire, and the rate of rupture-associated mortality is 2–3 times higher than that for AAA patients. While AAA is more commonly found in elders, TAA can occur at a young age due to the strong hereditary influence^59^.

Similar to AAA, no causal relationship has been found between atherosclerosis and TAA^60^, and it is relatively less associated with ascending TAA^55^. TAA often results from genetic disorders, and is now referred to as syndromic or familial TAA. In a study of 135 non-syndromic TAA patients without Marfan syndrome (MFS), almost 19% of the patients were found to have a family history of TAA and developed the disease in relatively a younger age^61^. Moreover, patients with a family history of TAA were found to have a faster aneurysm growth rate (0.22 cm/year) compared to patients with sporadic TAA (0.03 cm/year) or patients with MFS (0.10 cm/year)^61^. The specific genetic mutations associated with TAA are discussed later in this review. TAA has also been linked to inflammation and inflammatory diseases. Syphilis was once considered as one of the most common causes of ascending TAA^55^, and although extremely rare nowadays, recent cases of syphilis aortitis and TAA have been reported^62^. Aortic arteritis, which includes Takayasu’s arteritis and giant-cell arteritis, refers to a group of chronic inflammatory diseases of unknown etiologies, and is associated with a high prevalence of TAA^63,64^.

Recent clinical studies with large scale populations have identified fluoroquinolones, a commonly prescribed class of antibiotics as a risk factor for aortic aneurysm^65,66^. Fluoroquinolone usage elevated the risk of aortic aneurysm or dissection by 2–3 folds compared with the nontreatment group^67,68^, and by 2-fold compared with other antibiotics such as amoxicillin^69^. Fluoroquinolones are believed to affect the synthesis and structural integrity of collagen in the aortic wall^68^, or their chelating properties could affect collagen cross-linking and metalloprotease activities^70,71^. In a mouse model of AAA, ciprofloxacin (a fluoroquinolone) treatment significantly reduced lysyl oxidase (LOX) expression and activity, and increased MMP expression and activity^72^. However, the exact biological mechanism of action for this class of antibiotics requires further investigation.

## Experimental models of aortic aneurysm

Understanding the cellular and molecular event during the initiation, maturation, and rupture of human aortic aneurysm holds the key to identifying molecule(s) that are critical in the pathogenesis of this disease. However, aneurysmal aorta specimens from patients are only available at the advanced stages of the disease and therefore are not suitable for identifying the initiating factors. As such, a number of experimental models of aortic aneurysm have been developed and utilized on various genetically modified mice to understand the molecular mechanism(s) underlying aortic aneurysm (Table 1). Most of these experimental models share some of the features of human aortic aneurysm, although they all fall short of exactly mimicking the full clinical characteristics of this disease in humans^3,73^. Nevertheless, these models have helped provide insight into understanding the disease pathogenesis and can be utilized for the possible discovery of novel therapies for aortic aneurysm.

**Table 1:** The impact of genetic alterations of ECM-related genes on aortic aneurysm

Mouse strain	Aneurysm model used	Phenotype	Ref.
*Mmp2*^−/−^	CaCl_2_-induced TAA, AAA	Protection against AAA, TAA	83,108
	Ang II infusion	Exacerbated aortic dilation, TAA	83
*Apoe*^−/−^/*Mmp3*^−/−^	Cholesterol-rich diet	Reduced aneurysm formation	110
*Mmp9*^−/−^	Intraluminal elastase-induced AAA	Protection against AAA	84
*Mmp12*^−/−^	CaCl_2_-induced AAA	Protection against AAA	97
Wild-type/*Mmp14*^−/−^ bone marrow chimera	CaCl_2_-induced AAA	Reduced formation of AAA	109
*Adamts1* transgenic/*Apoe*^−/−^	Cholesterol-rich diet and Ang II infusion	No changes observed	115
*Adamts4*^−/−^	High-fat diet and Ang II infusion	Reduced aneurysm, dissection, and aortic rupture	98
*Adamts5*^Δcat^ (Truncated catalytic domain)	Ang II infusion	Increased dilatation of the ascending aorta	114
SMC- or EC-specific *Adam17*^−/−^	Adventitial elastase-induced TAA	Protection against TAA	88
SMC-specific *Adam17*^−/−^	Ang II + BAPN	Protection against TAA	116
*Apoe*^−/−^/*Cats*^−/−^	Ang II infusion	Protection against AAA	119
*Catk*^−/−^	Intraluminal elastase-induced AAA	Reduced AAA	120
*Catl*^−/−^	Intraluminal elastase-induced AAA	Reduced AAA	121
*Apoe*^−/−^/*Plat*^−/−^	High cholesterol diet	No changes observed	125
*Apoe*^−/−^/*Plau*^−/−^	High cholesterol diet	Protected against media destruction and aneurysm	125
*Plg*^−/−^	CaCl_2_-induced AAA	Reduced AAA	126
SMC-specific *Pai1* overexpression	Aortic xenograft	Prevented AAA formation	127
*Apoe*^−/−^/*Gzmb*^−/−^	Ang II-induced AAA	Protection against AAA	129
*Timp1*^−/−^	Intraluminal elastase-induced AAA	Enhanced aneurysm	131
*Timp1*^−/−^	CaCl_2_-induced TAA	Enhanced aneurysm	132
SMC-specific *Timp1* overexpression	Aortic xenograft AAA	Prevented AAA degeneration and rupture	133
*Timp2*^−/−^	CaCl_2_-induced AAA	Protection against AAA	137
*Timp3*^−/−^	Ang II infusion	Enhanced AAA	82
*Timp3*^−/−^/*Mmp2*^−/−^	Ang II infusion	Exacerbated AAA	82
*Eln*^−/−^	Spontaneous	Perinatal death	143
*Eln*^+/-^	Spontaneous	Supravalvular aortic stenosis	143
*Fbln4*^−/−^	Spontaneous	Perinatal death due to abolished elastogenesis	180
SMC-specific *Fbln4*^−/−^	Spontaneous	Spontaneous ascending and thoracic aortic aneurysm	181
*Fbn1* mutant	Spontaneous	Arteriopathy, aneurysm, and dissection	182
*Col1a1* ^Δ/ Δ^ (first intron deleted)	Spontaneous	Age-dependent aortic dissection and rupture	145
*Col3a1*^*+/−*^	Spontaneous	Sudden death due to TAA, dissection, and rupture	146
*Lox*^Mut/Mut^	Spontaneous	Spontaneous death after birth due to aortic rupture	149
*Bgn*^−/−^	Spontaneous	Spontaneous aortic dissection and rupture	18
*Apoe*^−/−^/*Dcn* overexpression	Ang II infusion	Reduced AAA and rupture	19
SMC-specific *Fbln4*^−/−^/*Thbs1*^−/−^	Spontaneous	Attenuated TAA compared with *Fbln4*^−/−^	21
*Thbs4*^−/−^	Ang II infusion	Attenuated TAA	159

*Fbln* fibulin, *Fbn* fibrillin, *Cat* cathepsin, *Bgn* biglycan, *Dcn* decorin, *Plat* total plasminogen activator, *Plau* urokinase-plasminogen activator, *Plg* plasminogen, *Pai* plasminogen activator inhibitor, *Gzmb* granzyme B, *Mmp* matrix metalloproteinase, *Apoe* apolipoprotein E, *Adam* a disintegrin and metalloproteinase, *Adamts* ADAMs with thrombospondin motifs, *Timp* tissue inhibitor of metalloproteinases, *Eln* elastin, *Col* collagen, *Lox* lysyl oxidase, *SMC* smooth muscle cell, *EC* endothelial cell, *Ang II* angiotensin II

### (i) Hyperlipidemic model

The hyperlipidemic model of aortic aneurysm utilizes subcutaneous infusion of a high dose of angiotensin II (Ang II, 1.5 mg/kg/d) in hyperlipidemic mice, namely mice lacking apolipoprotein E (*Apoe*^*−/−*^) or low-density lipoprotein receptor (*Ldlr*^*−/−*^) receiving a high-fat diet^73,74^. Long-term high-fat diet (HFD) feeding induces aortic inflammation and atherosclerosis in these mice^75^. This model is associated with medial phagocyte accumulation, medial dissection, elastic network degradation, and inflammatory cell infiltration^76^. The hyperlipidemic models of AAA share some features of obesity-induced vascular inflammation, atherosclerosis, and aortic aneurysm in humans.

### (ii) Angiotensin II infusion model

Subcutaneous infusion of Ang II, in the absence of hyperlipidemia, is commonly used in mice and rats to induce cardiovascular remodeling^77^. This relatively easy and minimally invasive surgical model mimics some features of human aortic aneurysm, including ECM remodeling, SMC activation, and vascular inflammation^73^. When used in combination with β-aminopropionitrile (BAPN), an inhibitor of collagen cross-linking enzyme LOX, Ang II infusion can result in the formation of TAA or AAA, or aortic dissection^78,79^. Simultaneous use of BAPN and a TGFβ inhibitor has also been reported to induce AAA in mice^80,81^. These aneurysm models display an aggressive development of intraluminal thrombus, elastin fragmentation, and influx of a range of inflammatory cells to the aortic wall^81^. Being a time-dependent progressive model, Ang II-induced aneurysm can be utilized for preventive and/or therapeutic studies. Intriguingly, Ang II infusion alone, in the absence of BAPN or hyperlipidemia, triggered AAA in *Timp3*^−/−^ mice^82^, and TAA in *Mmp2*^−/−^ mice^83^. As discussed in later sections, these two genetic models exhibit the importance of ECM-regulatory proteins in sustaining the homeostasis of aortic wall structure and the regional susceptibility of the aorta to aneurysm formation.

### (iii) Intraluminal elastase model

Intraluminal elastase perfusion model introduces porcine pancreatic elastase (PPE, 0.45 U/ml) into the infrarenal aortic lumen (at 100 mmHg, 5 min), which results in AAA formation within 2 weeks^84^. This model combines the mechanical strain with the enzymatic degradation of the elastic network in the aortic wall by PPE to induce injury, inflammation, and ultimately aneurysm^85^. This model is technically challenging as it requires the infrarenal aorta to be surgically separated from the inferior vena cava, and the lumbar and the juxtarenal arteries and the aortic bifurcation need to be temporarily ligated before the infrarenal aorta is punctured and infused with PPE^3^. As such, this model can only be used to induce AAA and not TAA since the close proximity of the thoracic aorta to the lungs and the heart drastically reduces the margin of error.

### (iv) Periadventitial elastase model

Periadventitial elastase model utilizes PPE to trigger degradation of the aortic elastic network by exposing the adventitia to elastase^86^. In this model, PPE (15 or 30 U/mL) is directly applied onto the adventitial surface of the aorta for 5 to 10 min to induce AAA^86^ or TAA^87,88^, respectively. This model is markedly less invasive than the intraluminal elastase model as it does not require aortic puncture nor pressure-induced mechanical damage. In addition, the adventitial elastase model can be used to induce AAA^86^ or TAA^87,88^. Although intraluminal and adventitial elastase models result in morphologically distinct aortic aneurysms in mice and thus represent specific subtypes of this disease in humans^89^, these aneurysms do not display thrombus, atherosclerosis, or rupture, which are classical features of human aortic aneurysm. A recent study showed that a combination of oral administration of BAPN and periadventitial elastase application induced a chronic, advanced-stage AAA with persistent growth, thrombus, and spontaneous rupture^80^. This represents a promising animal model to better understand the pathogenesis of AAA and to test promising therapeutic compounds.

### (v) Calcium chloride or calcium phosphate model

This model requires application of concentrated calcium chloride (CaCl_2_)^90^ or calcium phosphate (CaCl_2_ and PBS)^85^ solutions onto the adventitial layer of the aorta to induce calcification, elastin degradation, and subsequently aneurysm formation in 4–8 weeks. This model is site-specific and can be used to induce AAA and TAA^83^. Of note, CaCl_2_-induced TAA can be associated with a high operative mortality rate due to pulmonary complications upon exposure to CaCl_2_. In addition, the severity of aortic aneurysm in this model can be relatively mild.

### (vi) Decellularized aortic xenograft model

This model involves surgical interspecies transplantation of a decellularized aorta segment, for instance from guinea pig to rat^91,92^. The underlying principle of this model is to differentiate the impact of the cellular component versus the ECM. In the absence of the histocompatibility complex and protease inhibitors that are synthesized by the cellular compartment of the aorta, the decellularized aortic xenograft becomes the target of immune reaction and proteolytic degradation of the ECM and progressive aortic dilation^92,93^. Similar to human AAA, this model develops intraluminal thrombus and aortic aneurysm and rupture^92^.

### (vii) Large animal models

Large animal models of TAA and AAA are less diverse and less prevalently used compared to the rodent models, and have been proven useful for preclinical testing of endovascular devices or surgical procedures, rather than aneurysm pathogenesis. Intraluminal PPE perfusion has also been used in dogs, which, similar to the rodent model, leads to disruption of the medial elastic lamellae and aneurysm formation^94^. However, this model requires high concentrations of elastase and aneurysm formation is inconsistent. To avoid these issues, intra-aortic elastase and collagenase perfusion combined with aortic balloon angioplasty has been used^95^. In a pig model of TAA, intra-adventitial injection of collagenase with periadventitial application of crystalline CaCl_2_ resulted in elastic lamellar degradation, decreased collagen content, and TAA formation after 3 weeks^96^.

In summary, although each experimental model represents select characteristics of aortic aneurysm in the context of associated comorbidity(ies), and therefore can be useful in understanding the pathology of this disease. However, the interpretation of the findings should be exclusively based on the model used since contradictory results can be observed when the same animals are subjected to different models. For example, *Mmp12*-deficiency in mice suppresses CaCl_2_-induced AAA formation^97^, but does not affect elastase-induced AAA formation^84^.

## ECM-regulatory proteins in aortic aneurysm

Aortic aneurysm involves dramatic changes in the structural components of the vasculature, due to SMC dysfunction, imbalance between proteinases and their inhibitors, and abnormal synthesis, accumulation, or degradation of ECM proteins^3^. Among different classes of proteases, metalloproteinases (MMPs, ADAMs, and ADAM-TSs)^83,88,98,99^, and cathepsins^100,101^ are important factors for pathogenesis of aneurysm, because of their involvement in cleavage of different ECM and non-ECM proteins. The availability of genetically modified mouse models has paved the way to understanding the role of the ECM and its remodeling, the importance of protease and their inhibitors, and the contribution of different proteoglycans and glycoproteins in aortic aneurysm.

## Matrix metalloproteinases

Multiple MMPs contribute to physiological aortic remodeling and their increased expression are well documented in both human and mouse aortic aneurysms^102^. Under physiological conditions, vascular SMCs (VSMC) are the major source of MMPs production in the aorta, which is amplified under inflammatory conditions leading to augmented expression of MMPs^103^. Inflammatory cells, such as neutrophils and macrophages, also contribute to MMP production which further promote ECM degradation and aneurysm formation. Neutrophils can produce MMP8 and MMP9^104,105^, whereas macrophages can secrete a larger number of MMPs, including MMP1, MMP3, MMP7, MMP9, and MMP12^106^. These MMPs can cleave collagen (MMP1, MMP2, MMP9, MMP13, and MT1-MMP^107^), elastin (MMP2, MMP9, and MMP12), proteoglycans and basement membrane proteins, leading to the degradation of aortic ECM and aneurysm formation^103^.

Studies on MMP-deficient mice have provided us with important insight into the role of specific MMPs in different models of aortic aneurysm. *Mmp2*-deficient mice showed reduced aortic aneurysm formations in CaCl_2_-induced TAA and AAA models^83,108^. However, in response to Ang II infusion, *Mmp2*-deficient mice developed severe TAA but not AAA^83^. The absence of MMP9 in mice attenuated AAA formations^84^; however, the protection was reversed by introducing wild-type macrophages in *Mmp9*^−/−^ mice, but not in *Mmp2*^*−/−*^ mice, highlighting the role of macrophage MMP9 and stromal MMP2 in the pathogenesis of AAA^108^. Even in the absence of MMP2 or MMP9, MT1-MMP can be a key regulatory factor to induce aortic aneurysm by promoting macrophage-dependent elastolytic activity in CaCl_2_-induced AAA^109^. *Apoe*^−/−^/*Mmp3*^−/−^ mice showed reduced aortic aneurysm formation despite persistence of atherosclerotic plaques compared with *Apoe*^−/−^ mice^110^. Loss of MMP12, also known as macrophage metalloelastase, attenuated aneurysm formation in CaCl_2_-induced AAA model^97^.

Although there is significant evidence that MMPs are upregulated in AAA and TAA, and MMP-deficiency often results in protection against aneurysm formation, MMP inhibition may not be an effective approach in treating aortic aneurysm. Consistently, broad-spectrum MMP inhibitors, such as doxycycline, were reported to have promising effects in treating small AAAs^111^. However, recent randomized trials reported no changes in AAA growth or in reducing the need for AAA repair or time to repair in patients receiving doxycycline^112^.

## Disintegrin and metalloproteinases (ADAMs, ADAM-TSs)

A number of ADAM-TSs, including ADAM-TS4, ADAM-TS5, and ADAM-TS7, have been reported to be involved in TAA in humans and in mouse models^98,113,114^. Mice expressing truncated ADAM-TS5 (lacking the catalytic domain) showed increased aortic dilation in Ang II-induced TAA compared to wild-type mice^114^. ADAM-TS4 deficiency reduced aortic dilation, aneurysm formation, dissection and aortic rupture in a mouse model of sporadic aortic aneurysm and dissection (AAD) induced by HFD and Ang II infusion^98^. However, ADAM-TS1 overexpressing *Apoe*^−/−^ mice subjected to HFD and Ang II infusion did not show elevated aneurysm response compared to *Apoe*^−/−^ mice^115^.

Compared with ADAM-TSs, the role of ADAMs in aortic aneurysm is less explored. The most studied ADAM, ADAM17, was found to be critically involved in aortic aneurysm development in both humans and mice^88^. SMC- or EC-specific deletion of ADAM17 protected the aorta from elastase-induced TAA formation^88^, whereas ADAM17 deficiency in SMCs provided protection against AAA triggered by Ang II + BAPN treatment^116^. ADAM10 is an important regulator for vascular remodelling and inhibition of ADAM10 mRNA with miR-103a prevented AAA formation in *Apoe*^−/−^ mice following HFD and Ang II treatment^117^.

## Cathepsins

Cathepsins are a group of serine, aspartic, and cysteine proteases, which contribute to vascular remodelling. Several cathepsins, including cathepsins A, D, S, K, and L are reported to be involved or upregulated in humans aortic aneurysmal tissues^100,101,118^. Cathepsin K and S are potent elastases that are produced by SMCs and macrophages in the aorta under inflammatory milieus. Deficiency of cathepsin S attenuated Ang II-induced AAA formation in *Apoe*^−/−^ mice^119^. Cathepsin K has been reported to contribute to CD4^+^ T-cell proliferation and SMC apoptosis since its deficiency attenuated the AAA in the intraluminal elastase infusion model in mice^120^. Cathepsin L knockout mice exhibited reduced lesions, in vitro macrophage and T-cell transmigration, and angiogenic responses upon intraluminal elastase-induced AAA development^121^.

## Other proteases

There are several other proteases that are important in aortic aneurysm formation. Chymase and plasminogen activators are serine proteases that are involved in activation of other proteases, including MMPs. Chymases, secreted mostly by mast cells, activate MMP9 in human AAAs^122^. In addition to converting plasminogen into plasmin, plasminogen activators can directly degrade ECM components, activate collagenases, and degranulate neutrophils to release MMPs and elastases^123,124^. Deficiency of urokinase-plasminogen activator, but not tissue-plasminogen activator suppressed aortic aneurysm formation in hypercholesteremic *Apoe*^−/−^ mice^125^. Plasminogen deficiency also attenuated CaCl_2_-induced AAA by targeting macrophage migration and MMP9 activation^126^. Alternatively, local overexpression of plasminogen activator inhibitor-1 (PAI-1) prevented aneurysm development and rupture in mice^127^. Granzyme B is a proapoptotic serine protease that is abundantly expressed in advanced human atherosclerotic lesions and may contribute to plaque instability^128^. Granzyme B contributes to AAA through an extracellular, perforin-independent mechanism involving ECM cleavage. The absence of Granzyme B decreased AAA formation and increase survival of *Apoe*^−/−^ mice upon Ang II infusion^129^.

## Tissue inhibitors of metalloproteinase (TIMP)

TIMPs regulate the proteolytic activities in the vascular wall by directly binding to and inhibiting different groups of metalloproteinases, including MMPs, ADAMs, and ADAM-TSs. Although there is a high degree of structural homology among the four members of the TIMP family, they exhibit varying substrate affinities, inhibitory efficiencies, and differential responses to pathological stimuli^130^. TIMP1 can inhibit most members of the MMP family except the membrane-type MMPs (MT-MMPs) and MMP19, and few ADAMs, such as ADAM10^130^. TIMP1 expression is reduced in aneurysmal tissues, and the absence of TIMP1 resulted in severe aneurysm in the intraluminal elastase-perfused AAA^131^ and CaCl_2_-induced TAA mouse models^132^. Local overexpression of TIMP1 prevented aortic aneurysm degeneration and rupture in a rat model^133^. TIMP2 which acts as an inhibitor for many MMPs^134^ and ADAM12^135^, also plays an important role in MMP2 activation in coordination with MT1-MMP^136^. TIMP2 deficiency resulted in protection against CaCl_2_-induced AAA in mice^137^, perhaps by inhibiting MMP2 activation. TIMP3 has the broadest range of substrates among TIMPs which includes most of the MMPs, many ADAMs (ADAM −10, −12, −17, −19, and −33) and ADAMTSs (ADAMTS-1, −2, −4, and −5)^28^. Moreover, TIMP3 is distinct from other TIMPs in being ECM-bound^138^. Increased expression of TIMP3 in aneurysmal aortic tissue suggests its protective role against the disease^139^, as the absence of TIMP3 promoted aortic dilation and aneurysm in Ang II-induced (non-hyperlipidemic) AAA^82^. TIMP4 can inhibit a number of MMPs and ADAM28^140^. Interestingly, the N-terminal domain of TIMP4, but not the full-length protein, can inhibit ADAM17^141^. The role of TIMP4 in aortic aneurysm is less explored. Increased TIMP4 expression has been reported in hyperhomocysteinemia-associated aortic aneurysm in humans and mice^142^.

## ECM proteins that are central to aneurysm formation

### Elastin

Elastin is one of the principle ECM molecules synthesized by SMCs in the aorta in response to mechanical stress or pressure, and is formed as a complex of elastin protein assembled on a microfibril platform. Elastic fibers provide the aortic wall with a unique ability to expand and recoil that is essential for optimal blood perfusion throughout the body. Fragmentation of elastic fibers is a common feature of aortic aneurysm independent from the initiating factors, in both humans and animal models. Deletion of the elastin gene (*Eln*) in mice resulted in an uncontrolled proliferation of SMCs and perinatal death due to high blood pressure and tortuous, stenotic arteries. While elastin haploinsufficiency (*Eln*^+/−^) improved survival, it resulted in supravalvular aortic stenosis^143^.

### Collagen

Fibrillar collagens type I and type III account for 80–90% of the total collagen present in the aorta, while collagens type IV, V, VI, and VII represent the remaining fraction of collagens. Collagen turnover is critical for vessel wall repair and regeneration. In addition to its contribution to the vascular structure and tensile strength, it can regulate cell proliferation through interacting with integrins^14^. In humans, higher levels of collagens type I/III and collagen cross-linking are reported in aneurysmal aortas^144^, as increased collagen can enhance arterial stiffness and susceptibility to dissection and rupture. On the other hand, decreased collagen content and cross-linking can weaken the aortic wall, leading to aneurysm formation and/or aortic dissection^144^. The disparity in collagen content might reflect different phases of aortic remodeling, with fibrosis occurring at the late phase of inflammation during vessel repair. This also highlights the importance of sustaining a balance in collagen content for optimal aortic structure and function. Deletion of the first intron of *Col1α1* gene resulted in an age-dependent aortic dissection and rupture due to gradual decrease in collagen content^145^. Haploinsufficiency of *Col3α1* caused aortic dissection in mice and a pathology similar to the vascular-type Ehlers–Danlos syndrome (VEDS)^146^. Appropriate cross-linking of collagen monomers by LOX is critical for collagen fibril formation^147^, as inactivation of LOX, through mutations or gene deletion, can lead to TAA, cardiovascular dysfunction, and perinatal death in mice^148–150^.

### Proteoglycans/glycoproteins

Proteoglycans and glycoproteins comprise the non-fibrillar fraction of the ECM, which fills the extracellular space that is not occupied by the fibrillar ECM, and interacts with various molecules (growth factors, cytokines, etc.) to mediate their sequestration within the ECM. Several proteoglycans and glycoproteins have been reported to be upregulated during aortic inflammation and aneurysms^151^. The proteoglycans in the aortic wall mainly include large proteoglycans, such as versicans and aggrecans, or SLRP such as decorin, lumican, biglycan, etc^152^. These proteins are also involved in developing the pericellular matrix and promoting proliferation and migration of VSMC^153^. Versican is present in the intimal and medial layers of the aorta and is mostly expressed at the external part of the medial layer during vascular inflammation^17,154^. TAA and dissections are reported to have massive aggrecan and versican accumulation^155^; however, the levels of cleaved versican (V0 isoform) are decreased^17^. Biglycan is expressed throughout the aortic wall and regulates collagen fibrillogenesis^156^. Biglycan knockout mice develop spontaneous aortic dissection and rupture^18^. Similar to biglycan, decorin promotes collagen assembly and can be cleaved by Granzyme B, which has been associated with aortic aneurysm formation in mice^128^. Moreover, recombinant decorin fusion protein expression in mice attenuates Ang II-induced AAA formation and rupture^19^.

Thrombospondins, a family of secreted glycoproteins with antiangiogenic functions, are highly expressed in the aortic wall under inflammatory conditions. Thrombospondin-1 is reported to negatively regulate cell adhesion, migration, proliferation, and angiogenesis^157^. SMC-specific deletion of thrombospondin-1 attenuates TAA formation by restoring the disruption of the elastic lamina-SMC connections and preserving the mechano-transduction in mice and humans^21^. Thrombospondin-4 regulates vascular inflammation and atherogenesis^158^, and its deficiency can lead to aortic aneurysm formation following 3 weeks of Ang II infusion^159^. Fibronectin is a prevalent glycoprotein in the aortic wall, and its expression has been reported to be elevated in aneurysmal aorta from patients with bicuspid aortic valve and tricuspid aortic valve stenosis^160^. Recent studies have reported that impaired splicing of fibronectin is associated with TAA formation^161^. Tenascins are glycoproteins highly expressed in the aorta. Loss of tenascin C led to AAD in a combined CaCl_2_ and Ang II infusion model in mice, which was linked to enhanced inflammation, impaired TGFβ signaling, and collagen synthesis^162^.

### Aortic aneurysm associated with mutations in ECM-regulatory genes in humans

Over the last few decades, clinical studies have revealed a growing connection between mutations in ECM proteins and aortic aneurysm in humans (Table 2). Many of these mutations are well known, as they underlie the heritable syndromic diseases such as MFS, Loeys–Dietz syndrome (LDS), and Ehlers–Danlos syndrome (EDS); others are mostly responsible for non-syndromic aneurysm-related disorders^163,164^. MFS is one of the most extensively studied autosomal dominant disorder of the connective tissues. MFS affects the skeletal, ocular, and cardiovascular systems and is often associated with the development of TAA^165^. The incidence frequency of MFS is around 1:5,000 and it is implicated in 3–5% of all aortic dissections^166^. MFS is the result of mutations of the fibrilin-1 gene (*Fbn1*). Fibrillin-1 is a large, ECM glycoprotein that serves as a structural component of calcium-binding microfibrils^167^ that is central to elastic fiber assembly. Mutation of *Fbn1* impairs elastic fiber structure and promotes dilatation of the aortic root and the ascending aorta in the affected patients, causing aortic dissection and hemorrhage which can lead to sudden deaths^165,167^. Fibrillin-2 (*Fbn2*) is mostly associated with aortic development, and plays a major role during early morphogenesis in directing elastic fiber assembly and is found preferentially in elastic tissues, such as the cartilage, the tunica medial layer of the aorta and along the bronchial tree^168^. Mutations in *Fbn2* cause congenital contractual arachnodactyly, an autosomal dominant syndrome associated with aortic root dilation and aneurysm^169^. Fibrillin proteins exhibit marked structural homology to latent TGFβ binding protein, and are believed to influence TGFβ signaling in MFS^163,170^. However, the importance of TGFβ signaling in aortic aneurysm is independently recognized in another human aortopathy, LDS. LDS is an autosomal dominant disorder characterized by aggressive aortic root and TAAs where multiple family members are responsible for different types of LDS^171^. All five LDS variations (LDS1-5) are characterized by respective mutations of the following genes, *Tgfbr1*, *Tgfbr2*, *Smad3*, *Tgfb2*, and *Tgfb3*. The mutations present in *Tgfbr1* and *Tgfbr2* are principally missense and mostly occur in the kinase domains of the receptors^172^.

**Table 2:** ECM protein mutations associated with hereditary aortic aneurysm in humans

ECM gene	Identified genetic alterations	Syndrome/phenotype	Ref.
*Col1α1*	Missense mutations	Osteogenesis imperfecta; Ehlers–Danlos syndrome type 7A	175
*Col1α2*	Missense mutations	Osteogenesis imperfecta; Ehlers–Danlos syndrome type 7B	175,183
*Col3α1*	Multi-exon deletions	Ehlers–Danlos syndrome, type 4	173,184
*Col4α1*	Mutations in exon 24, exon 25	Hereditary angiopathy, nephropathy, and aneurysms	177
*Col4α5*	Nonsense mutations	X-linked Alport syndrome; TAA, AAA	178
*Eln*	Multiple point mutations	Supravalvular aortic stenosis; ascending aortic aneurysm and dissection	179,185
*Fbn1*	Missense mutations	Marfan syndrome; TAA	186
*Fbn2*	Mutations of intron 32, resulting in missplicing of exon 32	Cutis laxa with aneurysm; TAA	169
*Fbln4*	Heterozygous mutations	Cutis laxa with aneurysm	187
*Lox1*	Loss-of-function missense mutation	TAA and dissection	149,150
*Smad3*	Mutations at the MH2 domain	Loeys–Dietz syndrome type 3	188
*Tgfβ2*	Heterozygous mutations or microdeletions	Loeys–Dietz syndrome type 4	189
*Tgfβr1*	Heterozygous mutations	Loeys–Dietz syndrome type 1	190
*Tgfβr2*	Heterozygous mutations	Loeys–Dietz syndrome type 2	190,191

VEDS is an autosomal dominant trait, which results from heterogenous mutation of type III procollagen gene (*Col3α1*) leading to frequent arterial dissections with TAA. VEDS is one of the most prevalent vasculopathies with an incidence rate of 1:5,000–20,000 in developed countries^173^. In the aorta, collagen supports and maintains the tensile strength and stiffness of the aorta for which collagen maturation and cross-linking are important. For proper assembly of collagen alpha mono-trimers into triple-helix collagen, it is important to have the repetitive presence of Gly–X–Y amino acid sequences in all three alpha chains. In VEDS, however, the glycine is replaced by other amino acids, which disrupts the formation of collagen alpha trimers and subsequently, intact collagen fibrils^174^. Individuals with VEDS are at risk of arterial rupture, aneurysm and dissection, uterine rupture during pregnancy, and gastrointestinal perforation or rupture which could cause sudden death^164^. Mutations in collagen genes can also induce aortic dysfunction. Mutations of *Col1α1* and *Col1α2* are reported in patients with osteogenesis imperfecta with rare aortic aneurysm^175^. However, *Col1α1* mutation has been associated with dissections in medium-sized arteries and *Col1α2* mutation with borderline aortic root enlargement with regurgitation in patients^175,176^. Collagen IV is a major component of the basement membrane in various tissues including the aorta, and mutation of *Col4α1* gene has been linked to hereditary angiopathies, including nephropathy and aneurysm^177^. Mutation of *Col4α5* is associated with X-linked Alport syndrome and ascending aortic and AAA and dissection^178^. A missense mutation in the *Lox* gene has been linked to TAA and aortic dissection because of insufficient elastin and collagen cross-linking in the aortic wall^149^.

Elastin is a key structural component in cardiovascular development, vascular elasticity and structural integrity of the aorta. Decreased elastin in humans is associated with development of an autosomal dominant disease, supravalvular aortic stenosis (SVAS). SVAS results from loss-of-function mutations in the elastin gene and is associated with aortic stenosis, hypertension, and cardiac failure^179^.

## Conclusion/future prospects

In conclusion, aortic aneurysm remains a serious health concern as its rupture can cause significant morbidity and mortality. It is well acknowledged that TAA is a distinct disease from AAA and should be treated as such. Research to date suggests that given the regional heterogeneity of the aortic structure and ECM, events that disrupt ECM synthesis or ECM protein assembly could underlie the formation of aneurysm in the thoracic area, whereas factors that trigger enhanced proteolytic degradation of the ECM contribute to AAA formation. This is consistent with the notion that genetic disorders that disrupt collagen or elastin production (or assembly) are associated mostly with TAA and to a smaller extent with AAA. While studies on genetically modified mice have revealed a number of potential molecular and cellular mechanisms that may contribute to TAA or AAA, the lack of availability of an effective pharmacological treatment indicates that we may not yet have the full picture of all the factors that contribute to aortic aneurysm. Identifying the initiating factors in aneurysm formation is key in developing a treatment strategy, since at later stages, to repair the severe damage on the aortic wall if possible, would require effective replenishment of ECM-producing cells and ensuring optimal assembly and organization of the newly synthesized ECM proteins.
